# Accuracy of upper airway volume measurements using different software products: a comparative analysis

**DOI:** 10.1093/dmfr/twaf023

**Published:** 2025-03-14

**Authors:** Muhammed Enes Naralan, Taha Emre Köse, Merve Gonca, Büşra Beşer Gül, Dilara Nil Günaçar

**Affiliations:** Department of Oral and Dentomaxillofacial Radiology, Faculty of Dentistry, Recep Tayyip Erdoğan University, Rize 53000, Türkiye; Department of Oral and Dentomaxillofacial Radiology, Faculty of Dentistry, Recep Tayyip Erdoğan University, Rize 53000, Türkiye; Department of Orthodontics, Faculty of Dentistry, Eskisehir Osmangazi University, Eskisehir, 26040, Türkiye; Department of Orthodontics, Faculty of Dentistry, Recep Tayyip Erdoğan University, Rize, 53000, Türkiye; Department of Oral and Dentomaxillofacial Radiology, Faculty of Dentistry, Recep Tayyip Erdoğan University, Rize 53000, Türkiye

**Keywords:** cone-beam computed tomography, diagnostic imaging, oropharynx, segmentation, volume measurement

## Abstract

**Objectives:**

This study aimed to evaluate the accuracy of airway volume measurements obtained from cone beam computed tomography (CBCT) images using various software programmes, with a focus on assessing the performance of NemoStudio compared to other tools. The estimated volumes were compared with the volume of the solid model’s cavity filled with water (gold standard).

**Methods:**

A single 3D-printed airway model was created based on CBCT data and scanned 10 times under identical conditions. Volume measurements were performed using semi-automatic segmentation in 4 software programmes (NemoStudio, NNT Viewer, ITK-SNAP, and 3D Slicer). The results were compared to the gold standard using repeated measures analysis of variance, Bland-Altman plots, and *post hoc* comparisons.

**Results:**

Nemo Studio demonstrated a systematic bias and higher variability compared to the gold standard, resulting in lower accuracy than the other software programmes. ITK-SNAP and 3D Slicer showed the highest agreement with the gold standard, while NNT Viewer also exhibited acceptable performance. Statistical analyses revealed significant differences in the accuracy of volume measurements among the software tools (*P* < .001). Bland-Altman plots highlighted Nemo Studio’s broader limits of agreement, emphasizing its deviation from the gold standard.

**Conclusion:**

Variability in airway volume measurement accuracy underscores the need for careful software selection and methodological standardization. Further refinement of segmentation algorithms is essential for improved consistency and reliability in clinical applications.

**Advances in knowledge:**

This study provides the first evaluation of NemoStudio’s volumetric accuracy for CBCT-based airway measurements, offering novel insights into software reliability and the impact of algorithm selection in clinical and academic settings.

## Introduction

The upper airway volume is a critical parameter from both clinical and academic viewpoints. Accurate volume measurements are crucial for understanding the anatomical and functional properties of the airway, particularly for diagnosing and treating conditions such as obstructive sleep apnoea. These measurements are vital for assessing airway obstructions, evaluating the effects of surgical interventions, and objectively assessing treatment outcomes.^1^

Cone beam computed tomography (CBCT) is frequently preferred for airway volume evaluation due to its low radiation dose and 3-dimensional imaging capability. This method provides advantages such as rapid imaging, cost-effectiveness, and effective control over patient positioning. Computed tomography is generally performed with the patient in a supine position, whereas CBCT allows imaging with the patient seated or standing. In standing scans, the natural state of soft tissues is preserved because of gravity, which enables airway assessments to reflect normal function more accurately.^2–6^ Studies evaluating the accuracy of CBCT have demonstrated its clinical acceptability for measuring upper airway volume.^7^

Airway volume calculations are generally based on segmentation techniques. Manual segmentation, which requires the user to individually mark each slice, is considered the most accurate and reliable segmentation method.^8–10^ However, its time-consuming nature makes it impractical for routine clinical applications. Given that semi-automatic segmentation methods are more widely used in clinical workflows, this study focuses on comparing the accuracy of different semi-automatic segmentation software tools rather than manual segmentation. While volumetric subtraction between manual and semi-automatic segmentation could theoretically be used to assess discrepancies, our primary objective is to evaluate the effectiveness of semi-automatic segmentation software, as these methods are more feasible for clinical use. In contrast, semi-automatic segmentation provides a faster and more practical alternative. However, the accuracy of segmentation is influenced by the selected threshold values and the software used. Consequently, measurement outcomes may vary across different software, potentially affecting clinical decisions.^11^

In this study, volume measurements were obtained from a 3D-printed model of the oropharynx region. Nemo Studio, a commercially available software widely used in orthodontic and maxillofacial applications, was included in this study to assess its volumetric accuracy for airway measurements. Despite its clinical popularity, no prior research has systematically validated its segmentation performance in airway volume analysis, making this study the first to evaluate its accuracy in comparison to established tools. This study aims to fill this gap by comparing Nemo Studio’s performance with other validated tools, such as ITK-SNAP and 3D Slicer.

The aim was to evaluate the accuracy of volume measurements performed using different software programmes and to identify the software that provides results closest to the actual volume.

## Methods

### Ethical approval

This study was approved by the Ethics Committee of Recep Tayyip Erdogan University Faculty of Medicine (Approval Number: 2023/207). It was conducted in accordance with the ethical principles outlined in the Declaration of Helsinki. Owing to its retrospective design, the requirement for individual informed consent was waived by the Ethics Committee. All procedures adhered strictly to scientific and ethical standards.

### Selection of CBCT image

A patient without systemic disease or any hard or soft tissue pathology evident in their CBCT images was selected from the archives of the Department of Oral and Maxillofacial Radiology, Recep Tayyip Erdogan University Faculty of Dentistry. The images were obtained using a NewTom VGI evo (Cefla, Verona, Italy) device under the following parameters: 15 × 12 cm field of view (FOV), 0.2 mm voxel size, 110 kV, and 6.79 mAs. The patient’s images were oriented with the hard palate parallel to the ground, the sagittal plane perpendicular to the ground, and the coronal and sagittal planes mutually perpendicular. After orientation, the images were exported without any post-processing.

### 3D model generation and imaging

In the Digital Imaging and Communications in Medicine (DICOM) images, the oropharynx was delineated with its superior boundary defined as the posterior edge of the hard palate and its inferior boundary as the uppermost point of the epiglottis. Segmentation was performed using ITK-SNAP software. A dental and maxillofacial radiology specialist manually segmented the oropharyngeal region, ensuring high precision in boundary delineation.

**Figure 1.**
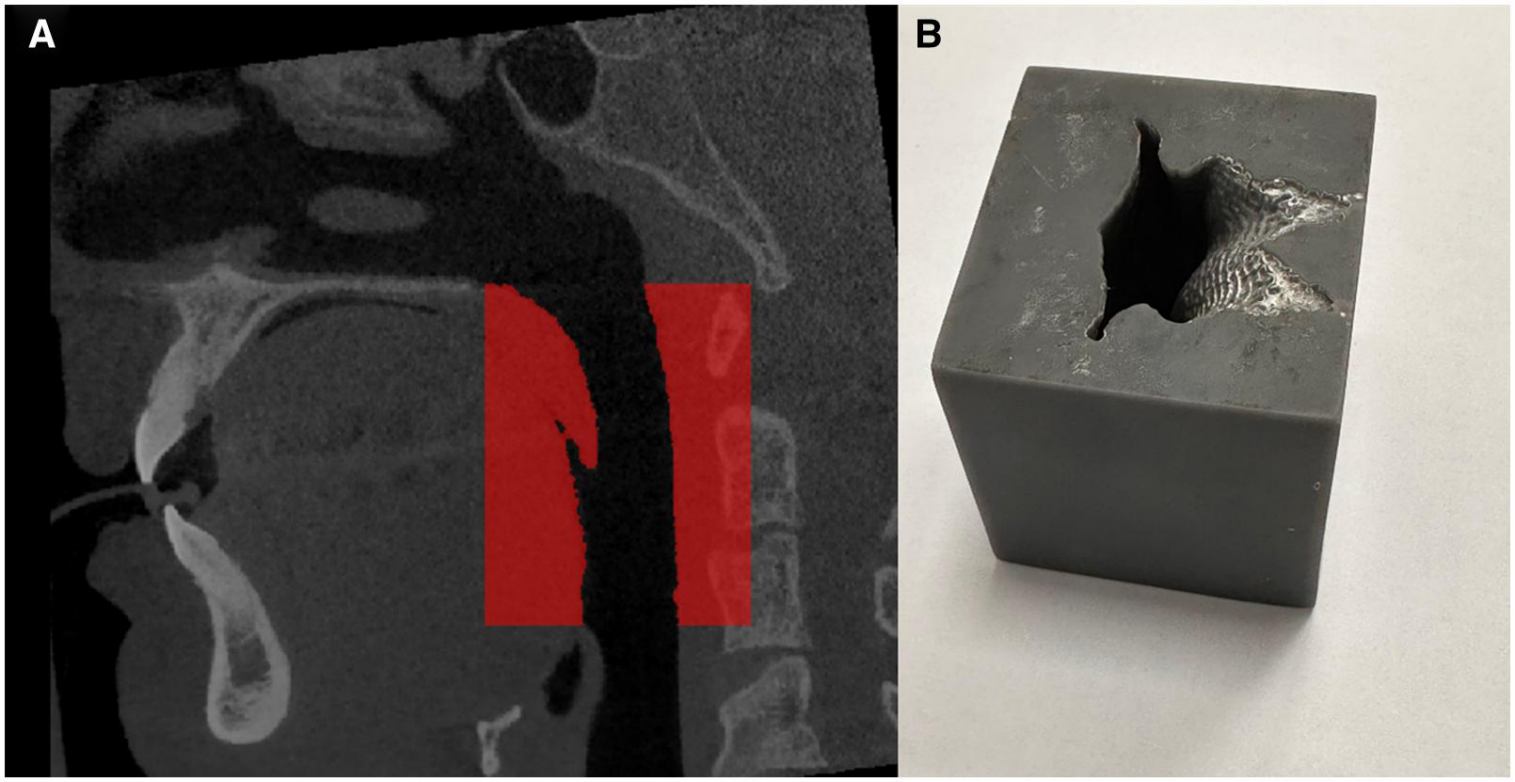
(A) Sagittal cone-beam computed tomography scan illustrating the segmentation region of interest (red area) used for volume analysis of the oropharyngeal region. The superior boundary is the posterior edge of the hard palate, and the inferior boundary is the uppermost point of the epiglottis. (B) The 3D-printed model of the oropharyngeal region, manufactured using a high-resolution resin printer, demonstrates the physical representation of the segmented volume for measurement and validation purposes.

The segmented region was exported in standard triangle language (STL) format and converted into a physical model using an Anycubic Photon Mono M7 Pro 3D printer (Anycubic, Shenzhen, China) with resin material. The printer was configured to a precision of 0.2 mm to capture the intricate details of the segmented region. This precision level was chosen to minimize geometric inaccuracies and ensure that the printed model accurately represented the original anatomical structures (Figure 1).

**Figure 2.**
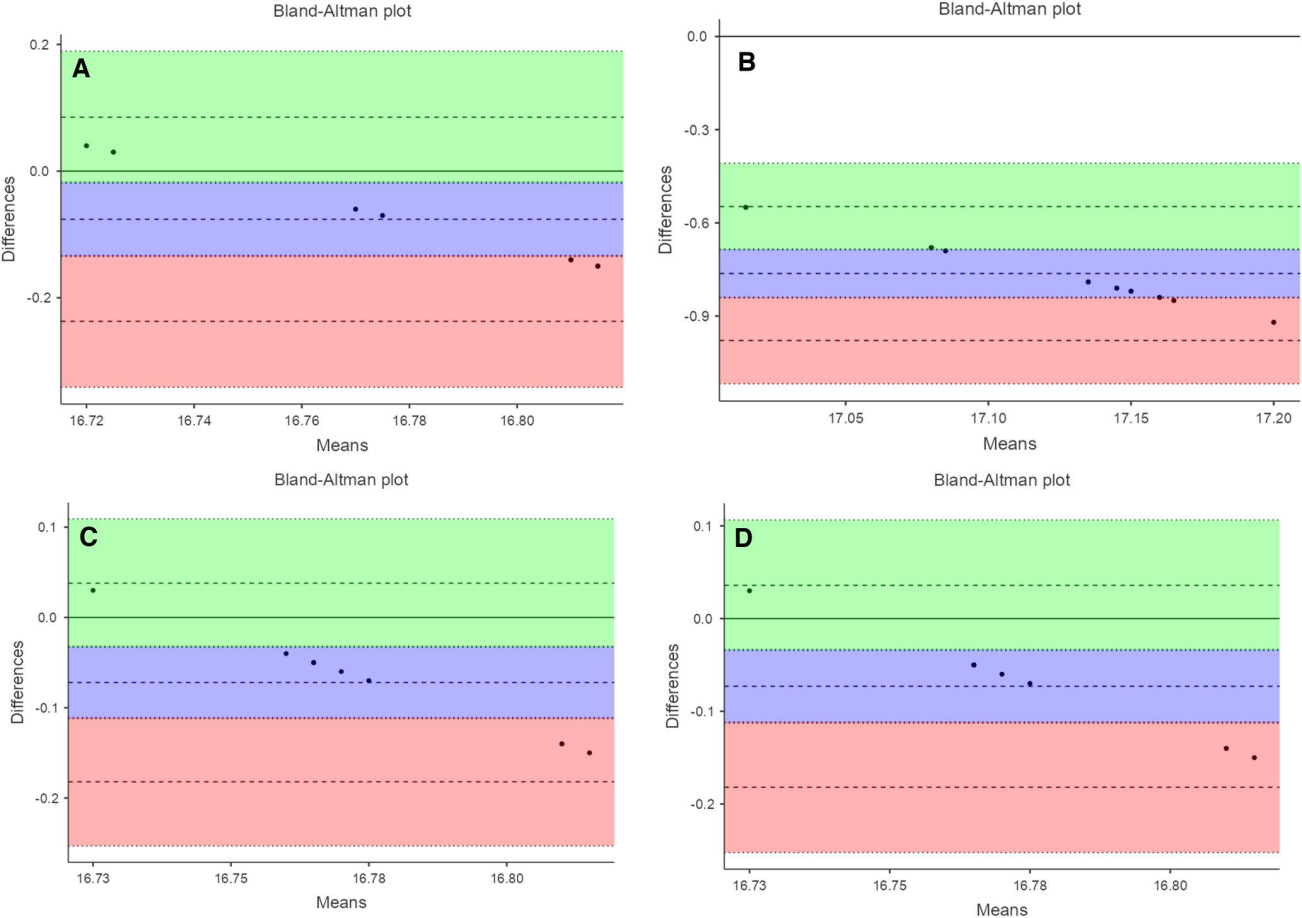
Bland-Altman plots comparing volume measurements between the software programmes and the gold standard. (A) Gold standard versus NNT Viewer, (B) gold standard versus NemoStudio, (C) gold standard versus ITK-Snap, and (D) gold standard versus 3D Slicer.

A single airway model was 3D printed and used as the reference standard. To ensure consistency and reliability in volumetric measurements, the model was scanned 10 times under identical imaging conditions. This approach minimized measurement variability, ensuring that observed differences stemmed from software performance rather than anatomical discrepancies.

Direct measurement of airway volume from the DICOM dataset may introduce bias due to software-specific segmentation variability. By utilizing a standardized 3D-printed model, we ensured that differences in measurements were solely attributed to software performance rather than anatomical variations. Therefore, the patient’s DICOM data were utilized solely to generate an anatomically accurate airway model, ensuring that the printed model faithfully represented real anatomical structures. The primary focus of this study was the airway volume in the 3D-printed model, as it provided a standardized reference for assessing software performance.

The model was scanned 10 times under identical conditions using the NewTom VGI evo device with fixed parameters: 8 × 8 cm FOV, 0.2 mm voxel size, 110 kV tube voltage, and 5.4 mAs exposure.

### Measurement of model volume

The volume of the solid model’s cavity served as the true reference standard (gold standard), allowing for a more objective evaluation of segmentation performance across different software tools. This approach minimized the influence of software-specific segmentation algorithms on the measurement and ensured a standardized comparison.

The 10 sequentially obtained images were exported in DICOM format without any post-processing. These images were then imported into the following software programmes:

- NNT Viewer version 13 (NewTom, Verona, Italy)
- ITK-SNAP (an open-source software developed through collaboration between the University of Pennsylvania Perelman School of Medicine and the University of Utah Scientific Computing and Imaging Institute)
- 3D Slicer (an open-source software developed in collaboration with Harvard University’s Brigham and Women’s Hospital and the Massachusetts Institute of Technology [MIT], supported by the U.S. National Institutes of Health [NIH])
- Nemostudio V 2020 (Nemotec, Madrid, Spain)

In all software programmes and for all images, semi-automatic segmentation was performed using the same threshold values: a lower limit of −1000 HU and an upper limit of −200 HU for the designated regions between the same slices.

The gold-standard measurement result was provided by using the volume of the cavity with water. The weight change method was employed to determine the cavity’s volume. Initially, the empty mass of the object was measured using a precision scale (Vibra Shinko AJ6200, Shinko Denshi Co., Ltd, Tokyo, Japan). The cavity was then filled with distilled water up to the reference point used for DICOM measurements and the filled mass was remeasured. The mass difference represented the mass of the water. By assuming the density of distilled water (*ρ*) to be 1 g/cm^3^, the cavity volume was calculated using the formula: volume (*V*) = (*m*_2_ − *m*_1_)/*ρ*.

The precision of the 3D printing process was also considered, with the selected printer matching the voxel resolution of the CBCT scans to minimize fabrication discrepancies. Furthermore, during the water-filling procedure, steps were taken to prevent air entrapment, including the use of a syringe at a 45° angle and vibration to eliminate residual air bubbles. A high-precision analytical balance was employed to ensure that only the mass of the water was recorded, preventing inaccuracies due to meniscus effects or trapped air. These methodological considerations were implemented to enhance measurement reproducibility and mitigate potential sources of error in airway volume quantification.

The results obtained were reviewed by a single author (M.E.N.) who blinded the dataset before providing it to the analyst (M.G.) performing the statistical evaluation.

### Statistical analysis

Statistical analyses were performed using SPSS version 21.0 (IBM SPSS, Armonk, NY, United States) and Jamovi version 2.2.5 (Microsoft Corp., Redmond, WA, United States). Descriptive statistics included measures such as the mean, SD, median, minimum, and maximum values. Data normality was assessed via histograms, normality curves, and the Shapiro-Wilk test.

A repeated measures analysis of variance (ANOVA) compared the gold standard with 4 software systems for volume measurements. Bonferroni correction was applied in *post hoc* pairwise comparisons to control the alpha error rate. A *P* value of <.05 was considered significant in all statistical evaluations.

Additionally, Bland-Altman plots were utilized to graphically assess the differences between the gold standard and software systems for volume measurements, providing insights into bias and agreement limits.

## Results

Descriptive statistics and the outcomes of the repeated measures ANOVAs are summarized in Table 1, while *post hoc* pairwise comparisons between each software system and the gold standard are detailed in Table 2. The repeated measures ANOVAs demonstrated significant differences among the 4 software systems and the gold standard (*P* < .001). *Post hoc* analysis highlighted notable variations in the average trueness of measurements across the software programmes, underscoring the differences in their alignment with the gold standard.

**Table 1.** Descriptive statistics of software programmes and the gold standard.

	Mean	SD	Median	Minimum	Maximum	*P*
Gold standard	16.74	0.00	16.74	16.74	16.74	<.01*
NNT	16.82	0.08	16.85	16.70	16.89	
NemoStudio	17.50	0.11	17.54	17.29	17.66	
ITK-Snap	16.81	0.06	16.80	16.71	16.89	
3D Slicer	16.81	0.06	16.80	16.71	16.89	

* The mean difference is significant at the .05 level.

**Table 2.** Pairwise comparisons between the gold standard and software systems.

(*I*) Factor1	(*J*) Factor1	Mean difference (*I*−*J*)	SE	Sig.^a^	95% CI for difference	
					Lower bound	Upper bound
Gold standard	NNT	−0.076	0.026	0.169	−0.172	0.02
	NemoStudio	−0.763^b^	0.035	<.001*	−0.891	−0.635
	ITK-Snap	−0.072^b^	0.018	0.029*	−0.137	−0.007
	3D Slicer	−0.073^b^	0.018	0.025*	−0.138	−0.008
NNT	Gold standard	0.076	0.026	0.169	−0.02	0.172
	NemoStudio	−0.687^b^	0.052	<.001*	−0.877	−0.497
	ITK-Snap	0.004	0.039	1.000	−0.138	0.146
	3D Slicer	0.003	0.038	1.000	−0.138	0.144
NemoStudio	Gold Standard	0.763^b^	0.035	<.001*	0.635	0.891
	NNT	0.687^b^	0.052	<.001*	0.497	0.877
	ITK-Snap	0.691^b^	0.026	<.001*	0.594	0.788
	D	0.690^b^	0.026	<.001*	0.595	0.785
ITK-Snap	Gold Standard	0.072^b^	0.018	0.029*	0.007	0.137
	NNT	−0.004	0.039	1.000	−0.146	0.138
	NemoStudio	−0.691^b^	0.026	<.001*	−0.788	−0.594
	3D Slicer	−0.001	0.001	1.000	−0.005	0.003
3D Slicer	Gold Standard	0.073^b^	0.018	0.025*	0.008	0.138
	NNT	−0.003	0.038	1.000	−0.144	0.138
	NemoStudio	−0.690^b^	0.026	<.001*	−0.785	−0.595
	ITK-Snap	0.001	0.001	1.000	−0.003	0.005

Based on estimated marginal means.

a Adjustment for multiple comparisons: Bonferroni.

* The mean difference is significant at the 0.05 level.

Among the analysed software programmes, ITK-Snap and 3D Slicer demonstrated the highest levels of agreement with the gold standard, with minimal bias and narrow variability. The NNT Viewer also exhibited strong agreement, though with slightly wider limits of agreement. On the other hand, NemoStudio showed a small but noticeable systematic difference and greater variability, indicating a relatively lower level of agreement with the gold standard.

Overall, the findings suggest that ITK-Snap and 3D Slicer are reliable alternatives to the gold standard for the evaluated parameters (Table 3).

**Table 3.** Bland-Altman plot for the consistency between the gold standard and software systems.

		Estimate	95% CI	
			Lower	Upper
Gold standard–NNT	Bias (*n* = 10)	−0.076	−0.1348	−0.0172
	Lower limit of agreement	−0.2371	−0.3413	−0.133
	Upper limit of agreement	0.0851	−0.019	0.1893
Gold standard–NemoStudio	Bias (*n* = 10)	−0.763	−0.842	−0.684
	Lower limit of agreement	−0.979	−1.118	−0.839
	Upper limit of agreement	−0.547	−0.687	−0.408
Gold standard–ITK-Snap	Bias (*n* = 10)	−0.072	−0.1122	−0.0318
	Lower limit of agreement	−0.182	−0.2531	−0.1109
	Upper limit of agreement	0.038	−0.0331	0.1091
Gold standard–3D Slicer	Bias (*n* = 10)	−0.073	−0.1128	−0.0332
	Lower limit of agreement	−0.182	−0.2524	−0.1116
	Upper limit of agreement	0.036	−0.0344	0.1064

The Bland-Altman plots (Figure 2) illustrate the differences between the gold standard and the software systems:

- X-axis represents the mean values of the gold standard and the software systems being compared, providing an average reference point for the data sets.
- Y-axis illustrates the differences between the gold standard and the software systems, offering a visual representation of the measurement discrepancies.
- Purple area (region of bias): highlights the degree of bias in the measurements.

The solid line within this area denotes zero bias, indicating perfect agreement.

The dashed line represents the estimated bias, showing the overall trend of deviation.

The dotted lines outline the 95% CIs for the bias, providing a statistical range for expected deviations.

- Green area (region for upper limit of agreement) defines the statistical boundary above which discrepancies are unlikely to occur.

The dashed line in this area marks the upper limit of agreement, calculated as the mean of the difference plus 1.96 times the SD of the differences.

The dotted lines specify the 95% CIs for this upper limit, adding precision to the estimation.

- Orange area (region for lower limit of agreement) represents the statistical boundary below which discrepancies are unlikely to occur.

The dashed line in this area signifies the lower limit of agreement, computed as the mean of the difference minus 1.96 times the SD of the differences.

The dotted lines indicate the 95% CIs for this lower limit, ensuring a comprehensive understanding of potential lower-bound deviations.

## Discussion

This study evaluated the effectiveness of different software programmes in measuring airway volume using CBCT data. The findings demonstrated that the accuracy of volumetric measurements varied significantly among the analysed software tools. These differences highlight the impact of segmentation algorithms and software-specific processing techniques on measurement reliability. Understanding these variations is essential for clinicians and researchers when selecting appropriate software for airway analysis, as discrepancies in volume estimations may influence diagnostic and treatment decisions.

CBCT is a widely used and well-established imaging technology for measuring airway volume, known for its accuracy and reliability in volumetric assessments.^1^^,^^12^ Its low radiation dose and rapid imaging capability help minimize errors caused by patient movement. Yamashina et al^6^ confirmed that CBCT provides measurements closely approximating actual airway volume, particularly in air spaces surrounded by soft tissues.^7^ Although CBCT has lower soft tissue resolution compared to other imaging modalities, this limitation does not significantly affect the accuracy of airway volume measurements.^6^

Several procedural factors were meticulously controlled to ensure the accuracy and reliability of airway volume measurements in this study. According to the literature, reorientation of the CBCT volume does not significantly impact measurement accuracy; however, it may slightly alter voxel distribution at airway boundaries.^13^^,^^14^ To address this, final volumetric assessments were conducted on the newly generated model post-reorientation, ensuring measurement consistency. Additionally, while STL conversion can introduce data loss,^15^^,^^16^ volumetric measurements were performed on the 3D-printed model rather than the digital STL file, thereby eliminating potential software- and operator-dependent errors associated with this process.

Random variations in radiation distribution and partial volume effects in CBCT can introduce inconsistencies in volumetric measurements.^12^^,^^17–19^ These effects occur when voxels contain multiple tissue types, leading to segmentation inaccuracies. Yamashina et al^6^ demonstrated that using multiple scans and averaging the results can help mitigate these errors. To minimize these effects, multiple CBCT scans of the same model were performed in this study, ensuring that measurement variations were reduced.

Positioning errors and device parameters are critical factors that can directly influence volumetric measurements obtained from CBCT images.^7^^,^^20^^,^^21^ In this study, the model was maintained in a fixed position, and all scans were performed using standard device settings.

Artefacts significantly contribute to variations in volume measurements.^19^^,^^22^^,^^23^ In this study, the use of a phantom minimized the effects of motion artefacts and positioning errors caused by patient movement.

Voxel size and threshold values in CBCT scans directly influence measurement accuracy.^24–27^ A study by Dong et al^24^ demonstrated that voxel size affects optimal threshold values, identifying 200 HU as the best minimum segmentation threshold for a voxel size of 0.2 mm. In this study, a threshold value of −200 HU was used to ensure accurate segmentation of the air-filled cavity.

This study identified significant differences in volumetric accuracy among the analysed software programmes. ITK-SNAP and 3D Slicer, validated open-source tools, are widely referenced in the literature.^28^^,^^29^ NNT Viewer, the default software provided with the CBCT device, is designed for ease of use, while Nemo Studio, a software, offers advanced orthodontic analysis capabilities. However, neither NNT Viewer nor Nemo Studio has been previously assessed for volumetric accuracy, making this study the first to evaluate Nemo Studio in this context.

In this study, Nemo Studio demonstrated lower accuracy compared to the other software programmes, while NNT Viewer produced results closer to the true measurement, following ITK-SNAP and 3D Slicer. These findings emphasize the need for further validation of software programmes like Nemo Studio, particularly in clinical applications where precise volumetric analysis is critical.

These findings suggest that different software solutions may not always provide the highest volumetric accuracy, as the segmentation algorithm plays a crucial role in measurement precision. In this study, volumetric measurements obtained using 4 different software programmes were compared to the weight difference method, which served as the reference gold standard for volume assessment. As expected, none of the evaluated software achieved perfect accuracy in representing the true volume. The observed discrepancies were primarily attributed to differences in segmentation algorithms, threshold values, and data processing methods. Similarly, a comparative study by Weissheimer et al,^11^ confirmed that all evaluated software programmes exhibited a certain degree of error. Their study also demonstrated that while none of the software tools were completely accurate, some produced measurements closer to the true volume than others.

The results of this study, along with findings from the literature, suggest that absolute volumetric accuracy cannot be fully achieved using current software tools, as measurement outcomes are inherently influenced by segmentation algorithms and processing techniques. However, previous studies have demonstrated that these programmes are highly effective in detecting relative changes in airway volume over time. For consistency, the same software should be used for serial assessments, as each programme employs unique algorithms that may affect comparative results.

The literature indicates that airway volume measurement accuracy is affected by user variability, segmentation algorithms, and methodological differences.^12^ Software, such as Nemo Studio, exhibited lower accuracy compared to open-source programmes like ITK-SNAP and 3D Slicer, which showed better agreement with the gold standard. However, even the most accurate software displayed minor but consistent deviations in volumetric measurements.^28^^,^^29^

As a limitation, minor variations may still occur due to anatomical differences and imaging-related factors. Volume averaging artefacts and X-ray attenuation can introduce slight inconsistencies, especially at the boundary between air and solid material. These effects may cause subtle fluctuations in gray-scale values, potentially influencing segmentation outcomes. To mitigate these factors, multiple scans were performed, allowing for a more robust assessment of measurement reliability and reducing the impact of random variations.

The results of this study emphasize the critical role of software selection in airway volume measurements, as accuracy is highly dependent on the algorithms and segmentation protocols used. Currently, no software achieves absolute measurement accuracy, highlighting the need for further refinement of segmentation techniques. To improve precision, more advanced algorithms must be developed, and segmentation protocols should be standardized. Additionally, cross-validation of results using multiple software tools in clinical applications may enhance measurement reliability and guide optimal software selection.
